# Foreign Summary

**Published:** 1838-08-01

**Authors:** 


### FOREIGN SUMMARY.

*Criminal Responsibility.—Venders of Quack Medicines, Quack Doctors, and Regular Practitioners.*—There are too many inconsistencies of all kinds in our patchwork laws and legislation to leave room for surprise at the manifest inconsistency of the portion of law on which we are now entering. This branch of law, like any other branch of criminal law on which there has been little legislation, is composed mainly of deductions from general principles of morals and convenience, made by the courts for the particular case or occasion. It is a branch of the common law, or, as it may not inaptly be called, our *unwritten wisdom;* and we may add, that on a subject like the present, we do not, in general, regret the absence of legislation; for such judge-made law is commonly more wise, more beneficent, and more just than the laws which emanate from parliament. Thus, in the case of quack medicines, parliament, instead of dishonouring, recognises the poisons, imposes a stamp, and with it a moral value, upon them; and their sale, with their impudent labels, and often fatally deceptive statements, become by necessary implication *legal,* and consequently dispunishable. But though the sale of quack medicines is legal, we beg to suggest to the medical corporations a distinction which we deem of some importance, and which will enable them to vindicate the rights of their members, and to protect the public health. The distinction rests upon the following principle:—That a man who sells poison under an attractive name commits a nuisance, and is indictable; he is indictable before any fatal consequence happens; the danger to the public makes the nuisance. The labels and statements which accompany the quack-poison are full of danger to ignorant persons, and it is a species of quackery which ought to be attempted to be put down by prosecution of the venders. The sale of these poisons could then be carried on only under the obscure name of the quacks themselves, without their delusive statements. A prosecution would establish the criminal responsibility of the simple vender of a poison, with a paper containing a recommendation of it to cases in which its use would be condemned by the whole body of the profession.

That there is great inconsistency in punishing persons who practise as physicians, surgeons, and apothecaries, without a license, and at the same time giving impunity to the sale of quack medicines, in the form in which it takes place at present, is evident, if we consider the real character of the bills which accompany the stamped poisons. What are they but *printed prescriptions* of particular doses of secret ingredients to be taken *as medicines?* What are they but substitutes for the prescriptions of the physician? Their very object is to render it unnecessary to resort to the educated judgment and skill of the apothecary, surgeon, or physician. This object they effect by inspiring the faith of the ignorant. The laws of parliament relating to the medical profession, and the charters of the medical bodies, establish the principle of protecting society in cases of disease against ignorance. There is the College of Physicians, to protect against ignorance in those who practise as physicians; the examiners of the Apothecaries' Company, to afford a similar protection with relation to that branch of the profession. No person can practise as a physician or apothecary without a license; and we affirm that upon a true construction of the 3 Hen. VIII., c. 11, it is illegal to practise as a surgeon without a license. Every portion of medical practice is thus embraced in the statute law, and the inconsistency is too great to be persisted in, of allowing the present mode of selling quack medicines, if it is attacked in the courts and in parliament, as it ought to be, by the medical corporations. There is, indeed, one circumstance in which the quack papers differ materially from a regular prescription;—that they are adapted, not for a particular case, or particular individual, but for all whose ignorance fits them for the imposition.

With respect to quack doctors, or unqualified persons,—for merely practising without doing harm, (if that is possible,) they are not responsible. Unlike quack papers, they have the power of discrimination; and as their *legal* inferiority only arises under the statutes and charters which establish the medical profession, they are liable only to the penalties provided against unqualified practisers in surgery and medicine. Their criminal responsibility therefore arises only in case of death happening through their want of skill or negligence.

It is an important question, whether they are entitled to a milder, or liable to a harsher consideration, in consequence of their not being members of the medical profession. In a civil point of view this ought to go in extenuation; if I prefer a person who has not the *legal* qualifications to one who has, I am not entitled to reproach him with his want of that skill which the law has secured to me in another person. But the public are not bound by this consideration; on the contrary, the public having already, in the provisions of different statutes, declared the importance of scientific qualifications, and having provided for them, may claim the exercise of extraordinary care and skill in those who practise without the legal qualifications; and, according to this principle, a quack doctor would have to bring to his patient at least the average knowledge, care, and judgment.

Such is the principle on which the criminal responsibility of quack doctors ought to be defined; but so rare are the cases which arise, so humane and kindly disposed generally are the judges, and so few the opportunities of discussing the principles of the criminal responsibility of this class of offenders, that it rather seems that this principle has been overlooked, and that quack doctors are held liable, like regular practitioners, only for *gross* unskilfulness or negligence. Thus, in the case of Webb, who was an agent for the sale of Morison's pills, and was indicted for having caused death by administering doses of gamboge, aloes, and other deleterious substances, one of the most learned and intellectual judges that ever sat on the bench (Lord Lyndhurst) told the jury, that if the death of the deceased was occasioned or accelerated by the medicines which the prisoner had administered, and that he had administered them either with a criminal intent, or *from gross ignorance*, they must find him guilty.*

The prisoner, Williamson, was seventy-five years of age, and had been in the habit of acting as accoucheur, though not regularly educated in that faculty. He was indicted for murder and manslaughter, in having caused the death of the deceased by mistaking a prolapsus uteri, which took place shortly after the delivery, for a remain of the placenta; and in endeavouring to remove the latter, he had lacerated the uterus, and torn open the mesenteric artery. This caused the death. Lord Ellenborough said, with reference to the charge of manslaughter, that, "to substantiate it, the prisoner must have been guilty of criminal misconduct, arising from the *grossest* ignorance or the most criminal inattention; one or other of these is necessary. It does not appear that there was any want of attention, and it appears that he had delivered many women at different times; from this he must have had some degree of skill. It would seem, that having placed himself in a dangerous situation he became shocked and confounded. I think that he could not possibly have committed such mistakes in the exercise of his unclouded faculties; and I own that it appears to me that, if you find the prisoner guilty of manslaughter, it will tend to encompass a most important and anxious profession with such dangers as would deter reflecting men from entering into it."

Of course the prisoner was found not guilty; though, in the opinion of the medical witnesses, the prisoner's mistake showed great want of anatomical knowledge.

* Rex v. Webb, 1 M. and R. 411.

In the case of Mr. Van Butchell, who was indicted for causing death by an injury in the rectum, caused by the introduction of a bougie, Mr. Baron Hullock broadly stated it to be his opinion, that "it makes no difference whether the party be a regular or an irregular surgeon;" and he adds, as a reason—"Indeed, in remote parts of the country, many persons would be left to die, if irregular surgeons were not allowed to practise;" and he adds, "There may be cases where both regular and irregular surgeons might be liable to indictment, as there may be cases where, from the manner of the operation, malice might be inferred."

This ruling, however, is opposed to an opinion in Lord Coke's Institutes, where it is said, "That if one who is not a regular surgeon take upon him to cure a man, and the patient die, this is felony;" which, though not literally correct, involves the principle which we have ventured to assert as the true principle of the criminal responsibility of quack doctors. As for the want of regular medical men in remote parts of the country, the surest way to exclude them is to establish a low principle of responsibility in the case of the irregular practitioners.

It is satisfactory to find more enlightened views taken in the case of Nancy Simpson, by Mr. Baron Bayley. A sailor discharged from the Liverpool Infirmary as cured, after undergoing salivation, went to the prisoner for an emetic to get the mercury out of his bones, and died from one dose of white vitriol, or corrosive sublimate, administered for this purpose by the prisoner. Mr. Baron Bayley said, "I take it to be quite clear, that if a person, not of medical education, in a case where professional aid might be obtained, undertakes to administer medicine which may have an injurious effect, and thereby occasions death, such person is guilty of manslaughter. He may have no evil intention, or may have a good one, but he has no right to hazard the consequence *in a case where medical assistance may be obtained.* If he does so, it is at his peril; it is immaterial whether the person administering the medicine prepares it, or gets it from another."

We have treated the subject of the criminal responsibility of quacks so fully, that our limits will not allow our dwelling on that of the regular practitioner; and it is scarcely desirable that we should do so, because we trust that a case can hardly happen of manslaughter by a regular practitioner; and what would be the rule of law in such a case, is sufficiently manifest in the preceding exposition relating to quack doctors.—*British Medical Almanac*, 1838.

---

*Practical Remarks on Poisons, by R. D. Thomson, M. D.*—When a medical man is called to see a patient who is suspected to have taken poison, or who complains of some sudden local pain of an irritating character, or is seized with some violent spasm or convulsion, his object should be first to make inquiries as to the probability of poison having been swallowed, and if the symptoms do not become mitigated, to form an immediate diagnosis. In preparing himself for this duty, it is necessary that he should be acquainted with the action of poisons in respect to the various structures which they attack, and to those parts of the body where the violence of their action elicits the most remarkable symptoms. The following observations are intended to simplify the subject, and to guide the practitioner by an attempt at a practical arrangement of poisons. When we analyze the symptoms produced by the action of poisons, we find that for practical purposes they may be divided into, 1. *Local Symptoms.*—Such as those produced by corrosive substances—acids, caustics, &c.; the poisons which produce these symptoms have usually been termed irritants, and constitute in toxicological works a large division of the subject of poisons. 2. *Spasmodic Symptoms.*—Where the action of the poison, although frequently exhibiting itself locally, extends generally to the nerves and muscles, and occasions violent spasms or convulsions, as strychnin, camphor, &c. 3. *Narcotic Symptoms.*—Where insensibility gradually supervenes after the poison has been received into the system, without any particular local or general derangement, as from the action of opium and other narcotics. 4. *Anti-spasmodic or paralytic Symptoms,*—as exemplified by the action of lead and manganese. In taking up each of these divisions *seriatim*, we shall merely dwell upon those points which concern the practitioner, omitting toxicological tests, a subject of too intricate and chemical a nature for any but a practical chemist to engage in, at least when the life of a fellow-creature is concerned. This investigation should be confided, as it is on the continent, to the care of experienced chemists. But with the symptoms produced by the agency of the various common poisons, and with the mode of relieving those symptoms, it is not only proper, but it is the duty of every medical man to make himself acquainted, and to have them so fixed in his memory that he may be enabled to apply his knowledge according to the exigency of the case.

I. Poisons producing local symptoms in the alimentary canal.—Irritant poisons. These are:—

MINERAL, OR INORGANIC.

| | |
|---|---|
| 1 Chlorine | 6 Potash, soda, alkaline carbonates |
| 2 Hydrochloric acid, nitric acid, sulphuric acid | 7 Nitrate of potash |
| 3 Iodine, and hydriodate of potash | 8 Lime |
| | 9 Mercury |
| 4 Phosphorus | 10 Bismuth, tin, gold, silver, zinc, antimony. |
| 5 Arsenic | |

VEGETABLE, OR ORGANIC.

| | |
|---|---|
| Castor oil | Colocynth, elaterium |
| Jatropha | Mezereon, aloes |
| Jalap | Euphorbium |
| Savin | Bryony. |

1. Chlorine gas.—*Symptoms.* In consequence of the great practical importance of this gas in the arts, it is not improbable that cases might occur where injurious effects may be produced upon workmen by its inspiration. The first symptom produced when chlorine is introduced into the trachea, is a feeling of roughness and constriction, which pervades the mucous membrane; coughing then supervenes, which is not accompanied by expectoration, if the lungs have been previously in a healthy condition. The glottis is more particularly the seat of the uneasy feeling of roughness and tickling; the chest experiences a sensation of oppression, and if the quantity inspired be considerable, the coughing continues for a long time, and the debility of body, at first progressing slowly, becomes in the end very great. I have seen a person, who had been employed in washing out bottles of chlorine over a water-trough, become so exceedingly exhausted as to be in a partial state of stupor, and to require several hours to walk the distance of a mile; the languor and total failure of strength and energy is so great, that it becomes almost impossible in some cases for the person even to walk. When given as an expectorant, therefore, great caution should be employed in its administration. *Treatment.* The object in the treatment is to neutralize the chlorine; this can be best effected by means of ammonia: all that is necessary is to breathe over caustic ammonia, or to inhale the odour of volatile carbonate of ammonia,—the consequence will be the formation of muriate of ammonia, which will not produce any deleterious effects upon the lungs. If chlorine water has been swallowed, a few drops of ammonia, in water, should be administered.

2. Mineral acids. *Hydrochloric*, *nitric*, and *sulphuric* acids.—*Symptoms.* When any of these acids is swallowed, a sense of violent heat is experienced along the œsophagus, and in the stomach, and then the symptoms of gastrodynia, or heartburn, appear; these increase in violence, accompanied with eructations, produced by the gases from the decomposition of the coats of the stomach; the matter vomited effervesces when it falls on the mortar of the floor, or when mixed with alkaline, carbonates, or limestone; the pain in the stomach becomes excruciating, the lips shrivel, the teeth become loose and yellow; great difficulty of swallowing is experienced, the pulse is weak and intermitting. *Treatment.* The action of these acids should be arrested by means of alkalis. Carbonates of soda or potash may be administered, in solution, or if these are not at hand, mortar or chalk may be diffused in water, and poured down or injected into the œsophagus. As great difficulty may be experienced in getting the patient to swallow, if the corrosion at the upper part of the gullet should have made considerable progress, anodynes and emollients should then be administered.

3. Iodine and hydriodate of potash.—*Symptoms.* These substances are not likely to cause deleterious effects, except in the form of overdoses of medicines, and therefore the practitioner will always be able to ascertain if they have been administered. Great pain and irritation is experienced in the stomach, vomiting supervenes, and when the quantity taken has been considerable, bloody diarrhœa occurs. The urine will probably exhibit the presence of iodine, when a solution of starch is added to it. *Treatment.* We are unacquainted with any antidote to iodine. Starch, which is a very delicate test for detecting its presence, forms a compound, whose action upon the animal economy has not been examined.

4. Phosphorus.—When this substance is swallowed, the symptoms are confined principally to the stomach, but so few cases have occurred, that they have not been well defined. The best antidotes are oils and ether.

5. Arsenious acid.—*Symptoms.* In the course of about half an hour after the poison has been swallowed, the patient is seized with sickness and great faintness; the stomach is affected with severe pain, which is increased by pressure; then follow vomiting, diarrhœa, and tenesmus. After these inflammatory symptoms have lasted for some time, spasms come on, the pulse sinks, delirium occasionally appears, and death takes place. If a white powder be observed in the matter vomited, a portion of it may be exposed to heat on the point of a knife; if it emit the odour of garlic, the probability is that it is arsenic. The mere occurrence of a powder does not necessarily imply the presence of poison; for I have seen oatmeal in the stomach of a person who had died suddenly, and had been exhumed on suspicion of having been poisoned, mistaken for arsenic; and therefore, if a powder occurs, it should be examined before any definite conclusion is drawn, unless for the benefit of the patient. Other symptoms distinguish the action of arsenic. *Treatment.* The first object is to accomplish the removal of the poison from the stomach; beyond which it must take a very considerable time to pass, in consequence of its great insolubility. If vomiting does not occur spontaneously, it should be excited by means of sulphates of zinc or copper, and copious libations of milk introduced into the stomach; the milk suspends the arsenious acid, and hinders it from acting on the coats of the stomach, and at the same time prevents the agony of dry vomiting. Gruel and barley water may be substituted for milk. This is merely to be the preliminary step while the following antidote is preparing:—Take a quantity of sulphate of iron, or green vitriol, dissolve it in water, and add a few drops of nitric acid, boil the mixture for a few minutes, and then add to it a solution of carbonate of soda; a bulky green precipitate immediately subsides; this is to be thrown on a filter, and washed with a little hot water; it is then to be diffused in water, and swallowed by the patient. The hydrous peroxide of iron combines with the arsenious acid, and forms a compound which is innocuous. For the discovery of this important antidote the profession is indebted to Dr. Bunsen, of Göttingen, and in fact it is the only antidote for arsenic with which we are acquainted. If sulphate of iron cannot be obtained, a substitute might be procured by boiling iron filings in a flask or pot with nitric acid, until a considerable portion is dissolved; the solution should then be precipitated by carbonate of soda as before. From the experiments of Dr. Bunsen upon dogs, it would appear that the administration of this antidote is quite effectual; if the poison has not remained too long in contact with the coats of the stomach, when it becomes embedded in the mucus, and probably enters into a partial combination with the animal matter.

6. Potash, soda, alkaline carbonates.—*Symptoms.* Violent pain in the region of the stomach; a burning pain along the internal surface of the œsophagus, vomiting, debility, slight spasms, hiccup, colic. *Treatment.* The alkalis should be immediately neutralized by means of vinegar or dilute acetic acid; oil has been recommended, but it is difficult to understand how oil, which usually requires heat and time to combine with alkalis, should be preferable to a weak acid whose action is instantaneous.

7. Nitrate of potash.—*Symptoms.* In doses of an ounce, pains of the stomach are experienced, dysentery supervenes, and death takes place, preceded by spasms. *Treatment.* We are not acquainted with any means of decomposing this salt in the stomach, and from its great solubility it will be impossible to arrest its action by any mechanical method. Anodynes may be administered with some benefit.

8. LIME.—*Symptoms.* Burning pain in the mouth and abdomen, thirst, and sometimes vomiting. *Treatment.* Acetic acid or vinegar; dilute mineral acids.

9. MERCURY.—*Symptoms.* When corrosive sublimate has been swallowed, the effects which follow are very similar to those of arsenic, but it differs in having a very disagreeable taste, of which arsenic is quite destitute,—in producing an immediate irritation of the throat; the stomach is the seat of a burning pain; nausea and vomiting occur, then diarrhœa, small pulse, general debility, cold sweats, cramps of the extremities, general insensibility, convulsions, death. *Treatment.* The best antidote is albumen. The whites of a number of eggs should be beat up with water, and administered until the symptoms are mitigated. M. Taddei has recommended a *powder*, consisting of five parts of fresh gluten and ten parts of soft soap, dried and ready for use. When triturated with water it forms a glutinous emulsion, which acts as an antidote to most mercurial preparations. Milk may be employed when the preceding cannot be obtained; iron filings have also been employed successfully.

10. BISMUTH, GOLD, TIN, and SILVER, act by corroding the surfaces to which they are applied, that is, by forming compounds with the animal matter. The symptoms must therefore be local, exhibiting themselves in the form of pain in the region of the stomach and intestinal canal, and increasing to spasms or convulsions, according to the severity of the dose. They are not likely to occur as poisons. The silver may be decomposed by common salt. Should sulphate or other salts of zinc be taken in an over-dose, the carbonate of soda, or ammonia, will act as an antidote.

11. ANTIMONY.—*Symptoms.* Vomiting, burning pain in the pit of the stomach, purging, colic pains, sense of tightness in the throat, violent cramps, delirium. *Treatment.* Encourage vomiting. A vegetable decoction should then be administered; as for example, decoction of bark, or bark in powder. Opium has been given with advantage after vomiting has ceased.

12. ORGANIC IRRITANTS.—The action of all vegetable and mineral purgatives depends upon their locally poisonous or irritating properties; when this action is restrained within proper bounds, a cathartic effect is the consequence; when it is pushed too far, the local irritation is so great that inflammation takes place; diarrhœa and dysentery follow, with faintness, convulsions, and death. The only treatment in these cases, is to employ mucilaginous and emollient drinks, and to have recourse to local bleeding if the irritation should warrant its application.

II. POISONS PRODUCING LOCAL AND SPASMODIC SYMPTOMS.

| | |
|---|---|
| 1 Carbonic acid | 8 Copper |
| 2 Ammonia | 9 Strychnin |
| 3 Cyanogen, hydrocyanic acid | 10 Camphor |
| 4 Oxalic acid | 11 Cocculus indicus |
| 5 Acetic acid | 12 Coriaria |
| 6 Sulphuretted hydrogen | 13 Poisonous fungi |
| 7 Barytes and strontian | 14 Cantharides. |

1. CARBONIC ACID.—*Symptoms.* The action of this gas, which is experienced in coal pits and mines of different kinds, is to irritate the nostrils and fauces so strongly as to cause the glottis to close spasmodically, and to prevent respiration of any kind from taking place. When it is disengaged in coal mines in particular situations, the workmen can only remain for a limited period; they are obliged to respire pure air at intervals; if they continue longer they are affected with weakness, giddiness, and a feeling of oppression over the head. When a bird is plunged into this gas it gasps, and its neck is thrown into convulsions, or at least moves to and fro, in a very remarkable manner. *Treatment.* The medical practitioner should inquire where the patient has been working; if it has been in a coal mine, or in the hold of a ship, it may be concluded that carbonic acid is the cause of symptoms similar to those described. The gas is liable to accumulate also in the excavations of limestone rocks, and in low valleys belonging to the same formation. The Grotto di Cane, and the celebrated Upas valley, in Java, owe their celebrity to the presence of this gas. The guides at the former locality are in the habit of exhibiting experiments on dogs, and of preventing them from suffering permanently, by an antidote, as appears from the following passage, extracted from "*Lithgow's Nineteen Years Travels*" in 1609.

Describing the occurrences at the Grotto di Cane, he says:—"The dog-keeper, for an easie composition, made triall of his dog; and having tyed a string to his hinder leg, he cast the dog scarce half way in the cave, where immediately his tongue hanging out, he fell downe dead. And forthwith his master repulling him back cast him in the lake, powring in water in his ears, but hee could never recover his life. Whereupon the poor man cryed out, 'Alas I am undone, what shall I doe, the dog that won my daily food is dead.'" It was obvious from this detail, that the method which the dog-keepers found most effectual in recovering their dogs from such accidents, was the administration of the cold affusion, or rather, the cold immersion. I have accordingly put this suggestion to the test of experiment, and have succeeded in perfectly recovering birds after immersion in carbonic acid, and when they were convulsed and apparently at the point of death, by plunging them once or twice into cold water. From this I am inclined to think that carbonic acid is not a poisonous gas, but acts by producing asphyxia. Bleeding has been generally recommended when the injurious effects of carbonic acid have been experienced, and as it has been strongly advised it should not be neglected, *but* the cold immersion should never be forgotten.

2. AMMONIA.—*Symptoms.* When ammonia or its carbonate is introduced into the system, severe pain of the throat and chest is experienced, with a degree of suffocation or insensibility, from the violence of the stimulation, and when the dose is considerable, convulsions are produced. *Treatment.* Muriatic acid in a dilute state, should be swallowed when the ammonia has been introduced into the stomach, and the fumes of the acid should be inhaled when the ammonia is acting deleteriously by its contact with the lungs.

3. CYANOGEN, HYDROCYANIC ACID.—*Symptoms.* According to the researches of Wöhler and Liebig, bitter almonds when distilled, are decomposed into an essential oil and prussic acid; prussic acid, however, does not exist either in almonds or laurel; but its basis, amygdalin, does, the oil and the prussic acid being formed by decomposition; this action appears to be readily excited by many agents; bitter almonds prove fatal when taken to a considerable extent, and therefore during digestion prussic acid would appear to be evolved. When a small dose of prussic acid has been taken, the symptoms are merely giddiness, slow pulse, and other symptoms of weakness. When the dose has been larger, however, these appearances are aggravated, and convulsions occur, which draw the head backwards; death then takes place without any other remarkable symptoms. *Treatment.* When prussic acid has been inhaled into the lungs or nostrils during distillation, or on smelling a strong solution, which is sometimes overpowering, ammonia should be immediately inhaled. Allow the person to smell liquor ammoniæ. If it has been taken internally, ammonia should be swallowed, diluted with water; when the symptoms come on gradually from the operation of a medium quantity, the cold affusion has been found very beneficial.—(*Edin. Med. and Surg. Journal*, July, 1837.) I have observed that the loss of blood greatly relieves the spasmodic symptoms. In a rabbit, whose carotid artery had been cut across and tied with a ligature, half a drachm of prussic acid of two and a half per cent., was inserted into an incision on the back; in about half a minute convulsions came on, and by their violence the ligature was forcibly removed; hæmorrhage of course took place, and the spasms immediately subsided; the death of the animal, which ensued, appeared to be occasioned rather by the loss of blood than by the action of the acid.

4. OXALIC ACID.—*Symptoms.* When the dose is concentrated, great pain is experienced in the stomach, and great debility of body with languor; and death takes place without spasms; but when the dose is more diluted, the pulse is low, the pain at the stomach either inconsiderable or absent; spasms at the chest come on; tetanus supervenes, and death seems to take place as if by suffocation. Vomiting generally accompanies the first attacks of pains in the stomach: the pain is of a burning nature. *Treatment.* The object is to neutralize the oxalic acid. This is best effected by means of lime; and this earth, in a state of solution, is to be preferred. Muriate of lime, if it can be had, should be administered in a dilute state; or acetate of lime; or lime water, which is more accessible. In the absence of these, magnesia or chalk suspended in water, by trituration, in a mortar, or by stirring with a rod, should be resorted to. As the action of the poison is very rapid, the practitioner cannot be too alert in the exhibition of the antidotes.

5. ACETIC ACID.—*Symptoms.* Pain in the stomach, vomiting, prostration of strength, convulsions, insensibility, death. *Treatment.* Neutralization of the acid by carbonate of soda, or potash, lime, or other alkali.

6. SULPHURETTED HYDROGEN.—*Symptoms.* This gas is exhaled from putrid vegetable and animal matter; and hence accidents may occur to workmen in common sewers, tunnels, &c. It is one of the most deleterious gases to animal life with which we are acquainted. When in a dilute state, it affects persons by producing speedily coma and convulsions, resembling those of tetanus, which extend to the trunk and extremities. When a bird is plunged into this gas, the chief symptoms are, convulsive efforts to breathe, retraction of the head, and rapid death. *Treatment.* Cold immersion, introduction of air into the lungs, respiration over a vessel filled with chloride of lime, and drinking chlorine water, free exposure to the air.

7. BARYTES AND STRONTIAN.—*Symptoms.* Burning sensation at the stomach, vomiting, convulsions, headach, deafness, death. *Treatment.* Dilute sulphuric acid; sulphates of soda, potash, magnesia, and other sulphates.

8. COPPER.—*Symptoms.* Pain in the stomach, coma, convulsions, tetanus, or great rigidity of muscles. *Treatment.* Sugar, albumen and iron filings, alkaline carbonates, ferrocyanate of potash.

9. STRYCHNIN.—*Symptoms.* The effects produced in a young girl by a pennyworth of nux vomica, in which strychnin is the active ingredient, as described by a nurse who saw her, were as follow:—She saw her seated in a chair, and said to her, "Why, Sarah, you appear drowsy." She answered, "I am;" then got up, and said, "Oh dear, how ill I am; I have taken poison." Afterwards she fell on the floor, struggled violently,—dreadfully,—was placed on the bed, threw up a large quantity of blood. Afterwards she became very quiet and still; the violent struggles were over; and then she emitted a deep sigh, which was her last. (*British Annals of Medicine*, vol. i. p. 105.) *Treatment.* It is always proper that the poison should be expelled from the stomach; this may be done, either by the stomach-pump, or by emetics. Orfila recommends the inflation of the lungs to prevent asphyxia. Donné has ascertained that in dogs the tincture of iodine, when administered in time, never fails to overcome the action of strychnin. Tincture of nut-galls has also been recommended; and M. Guimont has seen nut-galls in powder milk and sugar cure a dog poisoned with nux vomica.

10. CAMPHOR.—*Symptoms.* A sensation of heat in the stomach, sweat, delirium, convulsions, coma. *Treatment.* Emetics.

(To be continued.)

---

*Cystoplasty.*—M. Jobert lately presented to the Royal Academy of Medicine a female whom he had cured of vesico-vaginal fistula by the following simple operation. The fistula was about an inch in diameter, and the consequence of difficult labour. M. Jobert having refreshed the edges of the fistula, dissected off a flap from the external labium, and united it by suture with the refreshed edges of the sore. The first attempt failed, but the second met with the most complete success.—*Med. Zeitung, from French Lancet.*

